# Novel Variant of Tickborne Encephalitis Virus, Russia

**DOI:** 10.3201/eid1310.070158

**Published:** 2007-10

**Authors:** Vladimir A. Ternovoi, Elena V. Protopopova, Eugene V. Chausov, Dmitry V. Novikov, Galina N. Leonova, Sergey V. Netesov, Valery B. Loktev

**Affiliations:** * State Research Center of Virology and Biotechnology “VECTOR,” Koltsovo, Novosibirsk Region, Russia; † Regional Clinical Hospital No. 1, Vladivostok, Russia; ‡ Institute of Epidemiology and Microbiology, Vladivostok, Russia

**Keywords:** Tickborne encephalitis virus, TBEV, flavivirus, genotyping, polyprotein sequence, human lethal cases, dispatch

## Abstract

We isolated a novel strain of tickborne encephalitis virus (TBEV), Glubinnoe/2004, from a patient with a fatal case in Russia. We sequenced the strain, whose landmark features included 57 amino acid substitutions and 5 modified cleavage sites. Phylogenetically, Glubinnoe/2004 is a novel variant that belongs to the Eastern type of TBEV.

Tickborne encephalitis virus (TBEV) was originally isolated in the Far Eastern region of Russia in 1937 (*1*). TBEV is defined as a species within the *Mammalian tickborne virus* group (genus *Flavivirus,* family *Flaviviridae*) (*2*). The TBEV species includes 3 subtypes, Far Eastern (previously RSSE), Siberian (previously West-Siberian), and Western European (previously Central European encephalitis [CEE]) viruses. Recently, taxonomic improvements were proposed, and TBEV were divided into 4 types: Western, Eastern, Turkish sheep, and Louping ill (*3*). TBEV has been found in nearly 30 countries in Europe and Asia (*4*), and ≈700 million persons live in areas (excluding the People’s Republic of China) where TBEV infection is endemic. The annual incidence of TBEV infection is estimated to be as many as 14,000 cases (*5*). Eleven thousand TBE cases occur annually in Russia, but only ≈150 cases are registered in Primorsky District, Russia (*6*). The Far Eastern subtype is considered to be the most pathogenic for humans, with a mortality rate of >20%. The Western European subtype is less virulent and lethal (*7**,**8*). The TBEV genome consists of a single-stranded, positive-sense RNA of ≈11,000 nt that encodes 3 structural and 7 nonstructural proteins (*9*). The differences in nucleotide sequences encoding protein E between subtypes of TBEV may reach 18%–19%; amino acid sequences are considerably more conserved (*10*). In 2004, a total of 76 confirmed TBE cases occurred in the spring-summer season in Primorsky District; 10 were fatal. We describe 1 case caused by a novel variant of TBEV.

## The Patient

A 15-year-old boy received 2 tick bites near the village of Glubinnoe, in the northern part of Primorsky District, on May 30 and June 1, 2004, respectively. He had not had any vaccinations against TBEV infection. The first prodromal symptoms developed on June 8. A high fever with strong headache and paresis of cervical muscles developed in the next 2 days. On June 11, the patient was transported by emergency airplane from a local hospital to the Regional Clinical Hospital No. 1 in Vladivostok, where high fever with pronounced meningeal symptoms and complete disorientation in place and time were observed. Coma and acute respiratory dysfunction due to paralysis of respiratory muscles developed the next day, and the patient was put on an automatic respirator. He died of acute cardiovascular insufficiency and heart failure on June 17.

Virus strain Glubinnoe/2004 was isolated from a brain sample from the patient by using pig kidney embryo (PKE) cells. One hundred microliters homogenized brain diluted 1:100 was applied onto PKE cells in minimal essential medium supplemented with 2% fetal calf serum. The cells were incubated at 37°C for 4 days, and cell culture supernatants were used for second passage on PKE cells. The virus for sequencing and immunologic experiments was purified from infected PKE cells on third passage by centrifugation on a sucrose gradient (*11*). The protocol of study was approved by the Institutional Review Board of the Institute of Epidemiology and Microbiology, Vladivostok. Handling of the infectious material was performed under Biosafety Level 3–4 conditions.

A panel of monoclonal antibodies (MAbs) to TBEV was used for ELISA as described earlier (*12*). Viral RNA was extracted by using a RIBO-sorb kit (InterLabService Inc., Moscow, Russia); then RNA was transcribed to cDNA and amplified by PCR. The purified cDNA fragment was used for sequencing in a Beckman CEQ2000XL sequencer (Beckman Coulter, Inc., Fullerton, CA, USA). Fifty primers were designed on the basis of the TBEV sequence (GenBank DQ989336), allowing ≈150-bp overlap between adjacent PCR fragments. Each PCR fragment was independently amplified and sequenced 3×. Sequences were aligned with ClustalX (*13*). Molecular data were statistically processed by using the program MEGA (*14*). The program PHYLIP version 3.57 (University of Washington, Seattle, WA, USA) or PUZZLE version 4.0.2 (University of Munich, Munich, Germany) was used for constructing a phylogenetic tree.

The nucleotide sequences of the viral isolates were compared with published complete polyprotein sequences of TBEV (Table 1). The complete coding sequence of Glubinnoe/2004 was 10,886 nt. We found profound differences in the nucleotide sequences between listed TBEV strains and the Glubinnoe/2004 strain. The identity with 2 typical Far Eastern strains, 205 and Sofjin-HO, was 95.2% and 94.9%, respectively (Table 1). The complete Glubinnoe/2004 nucleotide sequence was compared with other TBEV sequences available in GenBank. The conservation of the strain’s nucleotide sequences ranged from 82.7% to 95.2%, whereas amino acid sequences’ conservation ranged from 91.0% to 98.4%, depending on the type of TBEV (Table 1). To further delineate the genetic variation, we analyzed polyprotein sequences of 37 other flaviviruses in comparison with Glubinnoe/2004 (Table 2). Fifty-three and 57 amino acid (aa) substitutions were found when Glubinnoe/2004 was compared to strains 205 and Sofjin-HO, respectively, and 14 of these were unique substitutions for all studied flaviviruses. Most substitutions were located in the C-terminal hydrophobic domain (CTHD) of proteins C, NS3, and NS5. The CTHD had 5 substitutions in 20 aa fragment; NS3, 10 substitutions, and NS5,16 substitutions. We also found that 5 putative cleavage sites of polyprotein were modified; the changes were located in viral C/CTHD and anchored C/prM sites. No substitutions were found in well-known features of protein E, such as the 12 cysteine residues, potential N-glycosylation sites, fusion peptide, and DEXH core motif of the NS3 helicase. A cysteine residue in position 4 of NS1 protein was replaced with a glycine; this mutation was described previously only for Sofjin-HO (BAB72162).

**Table 1:** Tickborne encephalitis virus (TBEV) strains used in the study and comparison of identify for full-length polyprotein sequences with strains Glubinnoe/2004 of TBEV*

Virus, subtype, strain	Place of origin	Year of isolation	Glubinnoe/2004	
			% Identity of nt sequence	% Identity of aa sequence
TBEV, Eastern type				
Glubinnoe/2004	Glubinnoe, Primorsky district, Russia	2004	100.0	100.0
205	Khabarovsk district, Russia	1973	95.2	98.4
Sofjin-HO	Khabarovsk district, Russia	1937	94.9	98.3
Oshima 5–10	Oshima, Japan	1995	94.7	98.2
Senzhang	China	1953	94.8	98.4
MDJ-01	China	2003†	94.6	98.0
Vasilchenko	Novosibirsk, Russia	1969	85.9	94.9
Zausaev	Tomsk, Russia	1985‡	85.9	94.9
EK-328	Estonia	1972	85.7	94.9
TBEV, Western type				
Neudoerfl	Neudoerfl, Austria	1971	84.3	93.4
263	Temelin, Czech Republic	1987	84.5	93.3
Hypr	Brno, Czech Republic	1953	84.1	93.0
TBEV, Turkish sheep type				
Turkish sheep encephalitis	Turkey	1969	82.9	91.0
TBEV, Louping ill type				
Louping ill, strain 369/T2	Selkirkshire, Scotland	1929	83.2	91.0
Spanish sheep encephalitis	Spain	1987	82.7	91.0

*TBEV type classification described previously (*3*). nt, nucleotide; aa, amino acid. †Time of submission of the sequence to GenBank. ‡Virus was isolated from a patient with chronic form of TBEV infection; a patient was infected in 1973 in Tomsk; the virus was isolated in Moscow in 1985 from brain.

**Table 2:** Individual amino acid substitutions of Glubinnoe/2004 and 205/Sofjin-HO viruses and unique substitutions in comparison with 37 flaviviruses, determined by polyprotein alignment*†

Gene	Amino acid substitutions Glubinnoe/2004 → 205 or Sofjin-HO		Unique substitutions	Changed putative cleavage sites	Summary of substitutions 205/Sofjin-HO
	205	Sofjin-HO			
Viral C	No	M_43_ → L; A_54_ → V; N_64_ → K	No	No	0/3
CTHD	A_99_‡ → V; I_108_ → V; M_111_ → I; M_113_‡ → V; F_115_‡ → L	A_99_‡ → V; I_108_ → V; M_111_ → L; M_113_‡ → V; F_115_‡ → L	No	Viral C/CTHD (RGKRR/SAA†DW) anchored C/prM (GM‡TF‡A/ATVRK)	5/5
prM	No	No	No	No	0/0
M	R_210_‡ → P	R_210_‡→ P; I_246_ → M; I_266_→ V	R_210_*	prM/M (SRTRR/SVLIR‡)	1/3
E	K_508_ → R; I_597_ → T; T_646_ → N; V_743_ → A	A_433_ → V; T_646_ → N; M_740_ → V; G_759_ → S	No	No	4/4
NS1	G_780_‡ → C; K_883_ → R; S_951_ → P; I_1053_ → T	I_1053_ → T; V_1122_ → I	No	E/NS1 **(**LGVGA/DVGG‡A)	4/2
NS2A	R_1180_ → K; R_1227_ → S; G_1250_ → S; G_1277_ → E; C_1311_ → Y	R_1180_ → K; R_1227_ → S; G_1250_ → S; G_1277_ → E; I_1296_ → T; A_1297_ → V	No	No	5/6
NS2B	V_1423_ → M	V_1423_ → M	No	No	1/1
NS3	E_1563_ → D; G_1650_ → E; I_1673_ → S; I_1707_ → T; T_1828_ → S; A_1861_ → V; P_1948_ → Q; V_1975_ → G; D_1988_ → N; A_2062_ → T	D_1491_‡ → G; S_1551_ → Y; E_1563_ → D; G_1650_ → E; I_1673_ → T; N_1731_ → S; T_1828_ → S; T_1869_ → A; P_1948_ → Q; V_1975_ → G; D_1988_ → N; A_2062_ → T	E_1563_; G_1650_; P_1948_; V_1975_; D_1988_	NS2B/NS3 (RTARR/SD‡LVF)	10/12
NS4A	D_2143_ → E ;V_2165_ → A	D_2143_ → E; A_2173_ → G	D_2143_	No	2/2
NS4B	M_2283_ → L; I_2314_ → M; A_2331_ → V; V_2349_ → I; A_2472_ → V	M_2283_ → V; F_2347_ → L; S_2457_ → A	M_2283_	No	5/3
NS5	K_2625_ → R; A_2757_ → G; G_2758_ → D; E_3013_ → G; S_3014_ → F; G_3033_ → E; K_3074_ → R; V_3080_ → I; S_3187_ → G; P_3251_ → R; V_3260_ → I; I_3297_ → V; V_3342_ → I; K_3389_ → E; Y_3402_ → D; N_3406_ → E	K_2526_ → R; M_2641_ → V; A_2757_ → G; G_2758_ → D; E_3013_ → G; Y_3030_ → H; V_3080_ → I; R_3188_ → G; P_3251_ → R; I_3297_ → V; V_3342_ → I; V_3343_ → A; R_3384_ → K; K_3389_ → E; Y_3402_ → D; N_3406_ → E	A_2757_; G_2758_; P_3251_; K_3389_; Y_3402_; N_3406_	No	16/16
Total			14	5	53/57

*Position number corresponds to the polyprotein sequence Glubinnoe/2004 of tickborne encephalitis virus (TBEV). CTHD, C-terminal hydrophobic domain. †The following polyprotein sequences were used for polyprotein alignment: DQ862460 (TBEV, Glubinnoe/2004); ABJ74160 (TBEV, 205); AAN73266 (TBEV, Senzhang); BAB71943 (TBEV, Oshima 5–10); BAB72162 (TBEV, Sofjin-HO); AF 069066 (TBEV, Vasilchenko);AAD34205 (TBEV, Vasilchenko); ABF46836 (TBEV, EK-238); AAO43537 (TBEV, Zausaev); NP_043135  (TBEV, Neudoerfl); AAZ80455 (TBEV, ts-variant of strain 263); AAA86739 (TBEV, 263); Q01299 (TBEV, Hypr); ABB90676 (Spanish sheep encephalitis virus); BB90675 (Turkish sheep encephalitis virus); AAQ91606 (OHFV, Bogolubovka); NP 878909 (OHFV, Bogoluvovska); AAR98531 (OHFV, Kubrin); ABB90677 (Greek goat encephalitis virus); NP_044677 (Louping ill virus);NP_620108 (Langat virus, TP21); AAF75260 (Langat virus, E5); P29837 (Langat virus, TP21);AAQ91607 (Kyasanur forest disease virus); NP_722551 (Alkhurma virus); NP_620099 (Powassan virus);AAL32169 (deer tick virus); ABB90669 (Gadgets Gully virus); ABB90671 (Karshi virus); YP_224133 (Karshi virus); ABE73208 (Karshi virus); ABB90673 (Royal Farm virus); ABB90670 (Kadam virus); ABB90674 (Saumarez Reef virus); ABB90668 (Meaban virus); ABB90672 (Tyuleniy virus); NP_689391). (Montana myotis leukoencephalitis virus). ‡For substitution in cleavage sites.

**Figure 1:**
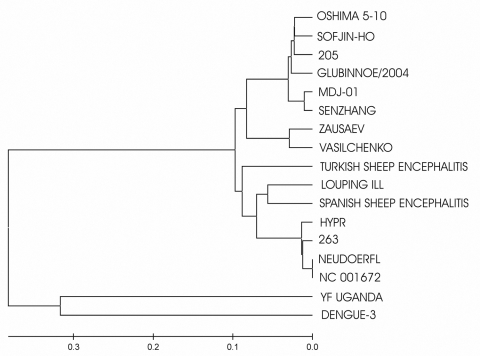
Phylogenetic tree, based on complete polyprotein sequences of tickborne encephalitis virus (TBEV). Original names of TBEV are presented. The multiple sequence alignments were obtained with ClustalX, and the tree was constructed by the neighbor-joining method. The following sequences were used for the phylogenetic tree: DQ862460 (TBEV, Glubinnoe/2004) ABJ74160 (TBEV, 205); AAN73266 (TBEV, Senzhang); BAB71943 (TBEV, Oshima 5–10); BAB72162 (TBEV, Sofjin-HO); AF069066 (TBEV, Vasilchenko); AAO43537 (TBEV, Zausaev); NP_043135 (TBEV, Neudoerfl); AAA86739 (TBEV, 263); Q01299 (TBEV, Hypr); ABB90676 (Spanish sheep encephalitis virus); BB90675 (Turkish sheep encephalitis virus); NP_044677 (Louping ill virus);NC_001672 (TBEV); AY217093 (TBEV, MDJ-01); AY968065 (yellow fever virus, Uganda); DQ675533 (dengue 3).

We also performed phylogenetic analysis of the fully sequenced TBEV strains and Glubinnoe/2004 (Figure 1) and analyzed 100 different protein E gene sequences available in GenBank (data not shown). Both phylogenetic trees clearly demonstrated that Glubinnoe/2004 belonged to the Eastern type and formed a separate clade (branch or group) within the type. The time of divergence of Glubinnoe/2004 from the Oshima-Sofjin and Senzhang groups was calculated by using the average substitution rate analysis (*15*). We estimate that Glubinnoe/2004 and Senzhang group diverged 300–470 years ago and Glubinnoe/2004 and Oshima-Sofjin group, 320–490 years ago. Our findings suggest that parallel evolution of different genetic groups of TBEV occurred in the relatively small Far Eastern region of Russia.

**Figure 2:**
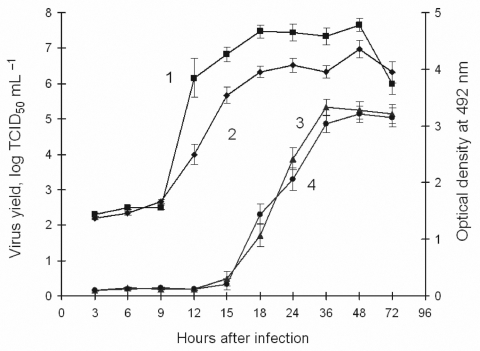
Growth curves and E protein synthesis of Glubinnoe/2004 and 205 in pig kidney embryo (PKE) cells: monolayer of PKE cells were infected with Glubinnoe/2004 and 205 of tickborne encephalitis virus (TBEV). The cells were frozen and thawed 3× to release the virus, and infectious titers were determined by the viral cytopathic effect (in 50% tissue culture infective doses [TCID_50_]/mL^–1^) assay in PKE cells. ELISA using 10H10 and biotin-labeled EB1 anti–protein E monoclonal antibodies was used to determine the E protein levels in virus-infected cells. 1, Glubinnoe/2004, virus yield; 2, 205, virus yield; 3, Glubinnoe/2004, E protein synthesis; 4, 205, E protein synthesis. Bars represent mean ± SD.

The growth curves for Glubinnoe/2004 and 205 viruses in PKE cells at 37°C are shown in Figure 2. The virus yield rapidly increased 9–18 h after infection and then stabilized. The maximum difference in virus yields (in 50% tissue culture infective doses [TCID_50_]/mL) between Glubinnoe/2004 and 205 viruses was 2.1 TCID_50_/mL at 12 h postinfection and nearly 10× from 15 to 36 h. By 72 h postinfection, PKE cells infected by Glubinnoe/2004 and 205 were completely lysed. We also evaluated the replication of viruses by directly measuring viral E protein levels by using 2 MAbs. E protein level rapidly increased 15–24 h postinfection and was similar for both strains. This finding correlated with a production rate of infectious virions with delay close to 6 h, which demonstrates that the Glubinnoe/2004 strain may produce more infectious virions in the early stage of infection of a PKE cell. The discovered mutations in 4 of 5 cleavage sites in viral polyprotein are required for cleavage of structural viral proteins. This finding may explain the more robust formation of infectious virions in the early stages of infection.

Serologic data demonstrate that strain Glubinnoe/2004 has epitopes well recognized by 14 anti-TBEV MAbs (data not shown). Based on these data, we concluded that all protein E epitopes of strain 205 virus are also present on Glubinnoe/2004 virions and are not affected by 4 aa substitutions found in protein E. This would suggest that modern vaccines against TBE and diagnostic immunologic kits will be effective against this novel variant.

## Conclusions

We have genetically characterized TBEV isolate Glubinnoe/2004 by determining its complete coding nucleotide sequence and comparing it with most of the available TBEV sequences. We found 53 and 57 aa substitutions in comparison with strains 205 and Sofjin-HO, respectively, and 14 of these were unique for 37 flaviviruses. Most substitutions were located in the CTHD of proteins C, NS3, and NS5. Phylogenetic analysis showed that Glubinnoe/2004 appears to be a separate lineage within the Eastern type of TBEV. We estimate that strain Glubinnoe/2004 diverged from Eastern TBEV in Senzhang group ≈300–470 years ago and from viruses in the Oshima-Sofjin group ≈320–490 years ago.

Five putative cleavage sites of the viral polyprotein were changed, including 4 sites responsible for processing of structural proteins. Strain Glubinnoe/2004 replicated more effectively in PKE cells than did strain 205. These data suggest that Glubinnoe/2004 could be used for production of vaccines, instead of strains 205 and Sofjin, and for development of diagnostic kits. This conclusion is supported by immunologic data with anti-TBEV MAbs, which demonstrated that viral glycoprotein E has a conserved antigenic structure typical of the Eastern type of TBEV.
